# Mechanism of Suppression of Chromosomal Instability by DNA Polymerase POLQ

**DOI:** 10.1371/journal.pgen.1004654

**Published:** 2014-10-02

**Authors:** Matthew J. Yousefzadeh, David W. Wyatt, Kei-ichi Takata, Yunxiang Mu, Sean C. Hensley, Junya Tomida, Göran O. Bylund, Sylvie Doublié, Erik Johansson, Dale A. Ramsden, Kevin M. McBride, Richard D. Wood

**Affiliations:** 1 Department of Molecular Carcinogenesis, The University of Texas MD Anderson Cancer Center, Smithville, Texas, United States of America; 2 The University of Texas Graduate School of Biomedical Sciences at Houston, Houston, Texas, United States of America; 3 Lineberger Comprehensive Cancer Center, Department of Biochemistry and Biophysics and Curriculum in Genetics and Molecular Biology, University of North Carolina at Chapel Hill, Chapel Hill, North Carolina, United States of America; 4 Department of Medical Biochemistry and Biophysics, Umeå University, Umeå, Sweden; 5 Department of Microbiology and Molecular Genetics, The University of Vermont, Burlington, Vermont; St Jude Children's Research Hospital, United States of America

## Abstract

Although a defect in the DNA polymerase POLQ leads to ionizing radiation sensitivity in mammalian cells, the relevant enzymatic pathway has not been identified. Here we define the specific mechanism by which POLQ restricts harmful DNA instability. Our experiments show that *Polq*-null murine cells are selectively hypersensitive to DNA strand breaking agents, and that damage resistance requires the DNA polymerase activity of POLQ. Using a DNA break end joining assay in cells, we monitored repair of DNA ends with long 3′ single-stranded overhangs. End joining events retaining much of the overhang were dependent on POLQ, and independent of Ku70. To analyze the repair function in more detail, we examined immunoglobulin class switch joining between DNA segments in antibody genes. POLQ participates in end joining of a DNA break during immunoglobulin class-switching, producing insertions of base pairs at the joins with homology to *IgH* switch-region sequences. Biochemical experiments with purified human POLQ protein revealed the mechanism generating the insertions during DNA end joining, relying on the unique ability of POLQ to extend DNA from minimally paired primers. DNA breaks at the *IgH* locus can sometimes join with breaks in *Myc*, creating a chromosome translocation. We found a marked increase in *Myc/IgH* translocations in *Polq-*defective mice, showing that POLQ suppresses genomic instability and genome rearrangements originating at DNA double-strand breaks. This work clearly defines a role and mechanism for mammalian POLQ in an alternative end joining pathway that suppresses the formation of chromosomal translocations. Our findings depart from the prevailing view that alternative end joining processes are generically translocation-prone.

## Author Summary

The reason for the hypersensitivity of POLQ-defective mammalian cells to ionizing radiation has been elusive. Here we show that POLQ-defective mammalian cells are selectively susceptible to double-strand breaks in DNA. We present experiments in mammalian cells showing that a specific double-strand break repair pathway is POLQ-dependent. To analyze the repair function in more detail, we examined class switch joining between DNA segments in antibody genes. Insertions of DNA bases are sometimes found at the joins between such segments, but the origin of these insertions has been mysterious. We show that this class of insertion joins during immunoglobulin class-switching is entirely POLQ-dependent. In experiments with purified human POLQ protein, we found a novel biochemical mechanism explaining the formation of the insertions. POLQ has a unique biochemical ability to extend DNA with minimal base pairing. Finally, we examined the biological consequences for chromosome stability. Unexpectedly, the Burkitt lymphoma translocation (a major cancer-associated genome instability) is enhanced in the absence of POLQ. This alters the current view about the action of DNA end joining in mammalian cells, revealing that a POLQ-dependent DNA repair pathway combats potentially damaging chromosome translocations.

## Introduction

A diverse group of at least 16 DNA polymerases carry out DNA replication, repair, and damage tolerance in the mammalian genome [1], [2]. One of these is DNA polymerase theta (POLQ). POLQ homologs are found in multicellular eukaryotes including plants, but an equivalent enzyme is absent from yeast [3]. The large 290 kDa human POLQ protein has an unusual bipartite structure with an N-terminal helicase-like domain and a C-terminal DNA polymerase domain [4]. This domain arrangement and the POLQ protein sequence is highly conserved in vertebrates [3].

Several functions have been suggested for POLQ [3] including bypass of template DNA lesions such as abasic sites and thymine glycols [5], [6], a backup role in DNA base excision repair [7], [8], and influencing the timing of DNA replication origin firing [9]. Loss of POLQ homologs in *Drosophila* and *C. elegans* causes hypersensitivity to DNA interstrand crosslink (ICL)-forming agents [10], [11] such as nitrogen mustards or cisplatin. A consistent picture of hypersensitivity to DNA damage in mammalian cells lacking POLQ has not emerged from studies reported so far [3]. Mice devoid of or carrying mutant alleles of *Polq* display an elevated level of micronuclei (indicating chromosome breaks) in their peripheral erythrocytes [12]–[14]. A further increased frequency of micronuclei is observed after ionizing radiation exposure, and is much elevated in *Polq* mutant animals [12], [14]. The majority (∼90%) of mice with double homozygous deficiencies in *Polq* and *Atm* die during the neonatal period, with surviving double mutant mice showing severe growth retardation [13]. From this observation it was suggested that POLQ has a unique role in maintaining genomic stability that is distinct from the major homologous DNA recombination pathway regulated by ATM [13].

DNA double-strand breaks (DSBs) can be formed in cellular genomes by environmental agents such as ionizing radiation. DSBs also arise during normal cellular duplication cycles, when DNA replication stalls at naturally occurring structures or at sites of internally-generated DNA damage. In diversification steps of the mammalian immune system, DSBs are deliberately formed by regulated enzymatic action, to initiate rearrangement of antibody and receptor segments, and as a means to introduce local variation. Because DSBs are toxic and/or mutagenic if not repaired, organisms have multiple mechanisms for DSB repair [15], [16]. The primary strategies are end-joining mechanisms, which process and rejoin the ends of a DSB, and homologous recombination (HR) pathways which employ an undamaged copy of the DNA [17]. End-joining pathways appear to be the first line of defense again DSBs. The most studied pathway is “classical” non-homologous end-joining (cNHEJ), which relies on the DNA-binding Ku70 (*XRCC6*) and Ku80 (*XRCC5*) gene products, and the DNA protein kinase (DNA-PK, *PRKDC*). One or more “alternative” end-joining pathways (altEJ) also exist, which are independent of these factors [18], [19]. During immunoglobulin diversification, the regional end-joining process of class switch recombination (CSR) replaces one constant region coding exon for another. This CSR process is known to occur through both cNHEJ and alternative end joining pathways [20]. In mammalian cells, an alternative end-joining repair pathway repair of DSBs is thought to play a role in driving the formation of chromosomal translocations, although the specific enzymology is unclear [21], [22].

Here, we report experiments that define a specific function and mechanism of action for POLQ in a pathway for alternative end-joining of DNA double-strand breaks in mammalian cells.

## Results

### Hypersensitivity to DNA strand-breaking agents in the absence of *Polq*

**Figure 1:**
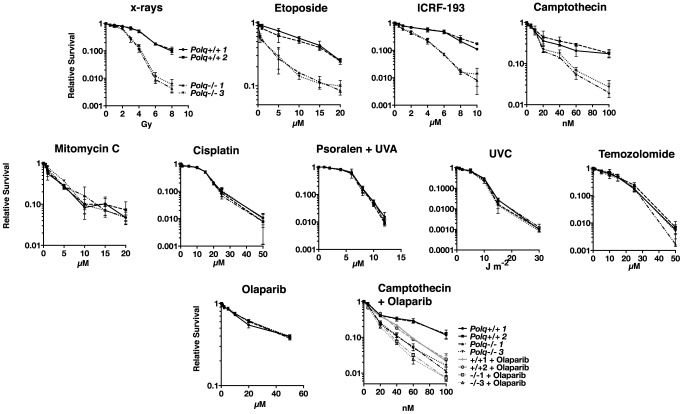
Hypersensitivity of *Polq^−/−^* bone marrow stromal cells to DNA strand-breaking agents. BMSCs were exposed to x-rays or UVC at the indicated doses, and to etoposide, ICRF-193, camptothecin, olaparib, temozolomide, mitomycin c, cisplatin, and HMT psoralen+UVA at the indicated concentrations and plated in triplicate. Two isogenic bone marrow stromal cell lines were used of each genotype, *Polq^+/+^* or *Polq^−/−^*. Colonies were crystal violet stained and counted seven to ten days later. Experiments were repeated three times. Circles, *Polq^+/+^* clone 1; Squares, *Polq^+/+^* clone 1; Triangles, *Polq^−/−^* clone 1; inverted triangles, *Polq^−/−^* clone 3.

To clarify the cellular role of POLQ in response to DNA damage, we measured the sensitivities of *Polq*-null and *Polq*-proficient bone marrow stromal cell (BMSC) lines to various DNA damaging agents. Cells lacking POLQ exhibit hypersensitivity to ionizing radiation (Figure 1) [12], [23], and to the double-strand break-inducing chemical bleomycin, as previously reported [12].

We found that *Polq^−/−^* cells were also hypersensitive to other agents which directly cause DNA breaks, including the topoisomerase II inhibitors etoposide and ICRF-193 [24] and camptothecin, a topoisomerase I inhibitor. In contrast, loss of *Polq* did not cause hypersensitivity to agents that largely form DNA replication-blocking adducts on one DNA strand including ultraviolet radiation and the methylating agent temozolomide. The *Polq^−/−^* cells were also not more sensitive than control cells to mitomycin C, cisplatin, and UVA-photoactivated psoralen plus UVA, all of which induce some interstrand DNA crosslinks (ICLs) (Figure 1).

These data indicate that POLQ is most important in a process conferring resistance to direct DNA strand-breaks, particularly double-strand breaks. Cells lacking *Polq* were not hypersensitive to the PARP inhibitor olaparib (Figure 1) while control RAD51D-defective cells were hypersensitive (Figure S1A). This suggests that POLQ does not function in the BRCA/homologous recombination pathway [25]. POLQ-proficient cells treated with both olaparib and camptothecin were significantly sensitized compared to camptothecin alone. However, addition of olaparib to *Polq*-null cells only modestly increased the sensitivity to camptothecin (Figure 1). Consequently, PARP and POLQ may operate within the same subpathway of DNA repair.

### Loss of *Polq* enhances chromosomal instability in somatic cells

**Figure 2:**
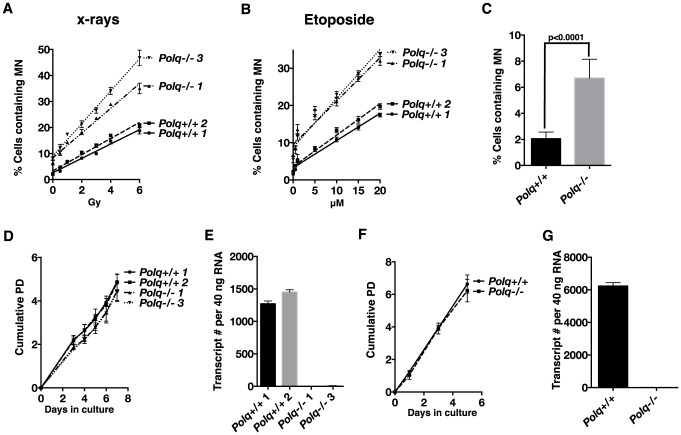
Loss of *Polq* contributes to chromosomal instability both spontaneously and in the presence of DNA damage. *Polq^+/+^* or *Polq^−/−^* bone marrow stromal cells plated in chamber slides were exposed to (A) X-rays or (B) etoposide. 48 hr after damage cells were fixed and stained with DAPI to enumerate cells with micronuclei. Counts represent the average percentage of cells with micronuclei scored in three independent experiments. (Slopes for X-ray and etoposide-induced MN: *Polq^+/+^* clone 1 (2.8, 0.73); *Polq^+/+^* clone 2 (3.1, 0.85); *Polq^−/−^* clone 1 (4.8, 1.2); *Polq^−/−^* clone 3 (6.2, 1.3). The frequency of spontaneous micronuclei for each of the clones in Figure 2A and 2B were combined to generate (C) total spontaneous micronuclei observed for all genotypes. The p-value was determined by Wilcoxon Mann Whitney rank sum test. *Polq^+/+^* or *Polq^−/−^* (D) bone marrow stromal cells and (F) mouse embryonic fibroblasts were plated in growth medium in triplicate. Cells were counted at the indicated days and cumulative population doublings were recorded. The experiment was repeated three times. (E and G) Absolute quantification of *Polq* transcript numbers in three independent experiments.

It is important to know whether the elevated level of micronuclei in *Polq*-defective cells extends to cell types other than peripheral erythrocytes. To answer this question, matched wild-type and *Polq^−/−^* BMSC lines were exposed to etoposide or x-rays, and the number of cells with micronuclei after 48 h were enumerated (Figure 2A and B). *Polq*-null cells exhibited a ∼3 fold increase in frequency of spontaneous micronuclei formation (Figure 2C). Upon exposure to DNA damaging agents, the percentage of cells with micronuclei increased about 1.5-fold more per unit of damage for *Polq^−/−^* cells in comparison to *Polq^+/+^* cells (Figure 2A and B). This shows that the susceptibility to micronucleus formation in *Polq^−/−^* cells is not confined to cells of the hematopoietic lineage, but occurs also in cultured cells, including fibroblast-like BMSCs.

Cells lacking *Polq* were analyzed for their ability to proliferate in culture. Two independent BMSC lines devoid of *Polq* expression proliferated at a rate comparable to a pair of wild-type control cells, the *Polq* BMSCs showing only a 5% increase in population doubling times (Figure 2D and E). We extended this analysis to isogenic immortalized mouse embryonic fibroblast (MEF) cell lines (Figure 2F and G). *Polq^−/−^* cells divided at a rate comparable to *Polq*-proficient cells. These findings fit with our previous observations that hematopoietic cell counts in irradiated *Polq*-null mice recovered at rates comparable to wild-type mice [12]. We have observed no major alterations in growth or development in unchallenged *Polq* null or mutant mice, consistent with previous reports [13], [14], [26]. These observations indicate that despite some increased chromosomal instability, POLQ-defective cells originating from a variety of tissues can proliferate at near-normal rates.

### The DNA polymerase activity of POLQ is required to confer resistance to DNA damaging agents

**Figure 3:**
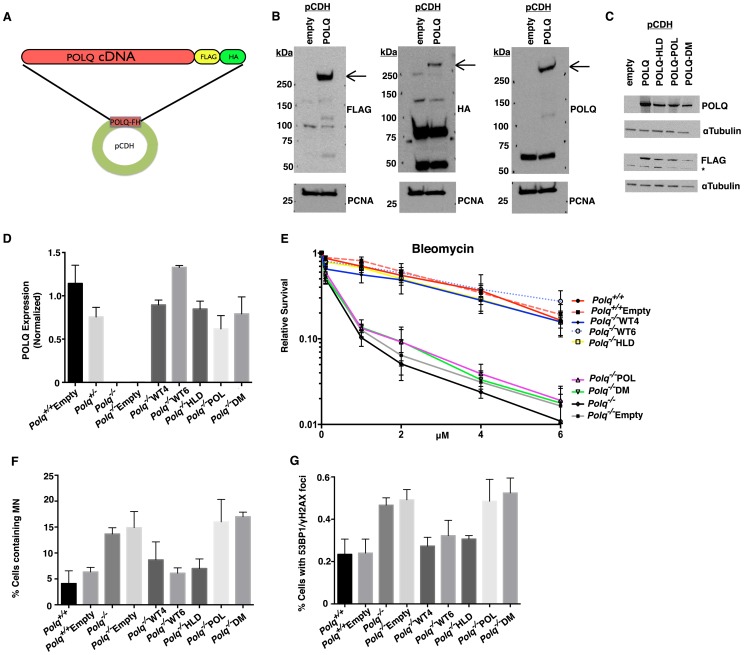
Complementation of the polymerase activity of POLQ rescues DNA damage hypersensitivity in cells lacking *Polq*. (A) POLQ cDNA was cloned into the pCDH-FH vector containing a FLAG-HA epitope tag on the c-terminus. 293T cells were transiently transfected with pCDH containing either empty-vector control or POLQ cDNA. (B) Crude extracts were immunoblotted with the indicated antibodies to confirm full-length expression of recombinant POLQ or (C) mutant constructs. (D) Stable MEF lines complemented with POLQ expression vectors (or empty vector control) were assayed for *Polq* expression by qPCR. WT4 and WT6 are independent clones complemented by wild-type POLQ; POL, mutation in the DNA polymerase domain; HLD, mutation in the DNA helicase domain, DM, mutation in both domains. (E) The complemented MEF lines were treated with bleomycin for 24 hr and cellular ATP levels were measured 72 hr later. (F) Spontaneous micronuclei and (G) DNA double-strand breaks (>2 colocalized γH2AX and 53BP1 foci per cell), quantified for three independent experiments. The brightness of the entire microscope field was increased to better display the fluorescence for publication, using Adobe Photoshop CS6.

We sought next to investigate which catalytic activities of POLQ are necessary to confer resistance to DNA damaging agents. Lentiviral-delivered expression vectors were constructed to express wild-type or mutant versions of POLQ in immortalized MEFs, in order to test for functional complementation (Figure 3A). A tandem (D2330A,Y2331A) mutation was introduced into the DNA polymerase domain (POL); mutation of the corresponding residues in other DNA polymerases completely inactivates polymerase activity [27]. In a separate construct, a mutation was introduced into the conserved ATP-binding site of the Walker A motif (K121M) in the helicase-like domain (HLD). An equivalent mutation eliminates DNA helicase activity in related enzymes, including HELQ [28]. A third construct (DM) was made harboring mutations in both domains. These vectors expressed full-length recombinant POLQ as tested in transfected 293T cells (Figure 3B and C).

The mutant cDNAs were tested for their ability to genetically complement the bleomycin sensitivity of *Polq*-null MEFs. Stable clones with each of the constructs were generated and analyzed for expression of POLQ (Figure 3D). Independent clones of knockout MEFs expressing wild-type recombinant POLQ (WT4 and WT6) were able to rescue bleomycin hypersensitivity (Figure 3E) as an antibody that recognizes endogenous POLQ does not yet exist. Neither the polymerase domain mutant (POL) nor the polymerase-helicase double mutant (DM) restored bleomycin sensitivity (Figure 3E, Figure S1B). Expression of a construct with a mutation only in the helicase-like domain (HLD) was, however, still able to restore resistance to bleomycin. These data indicate that POLQ polymerase activity is essential for conferring resistance to DNA damage, while the ATPase activity of the helicase-like domain is not necessary. Similarly reintroduction of polymerase activity of POLQ into *Polq*-deficient MEFs was able to rescue chromosomal instability (micronuclei and DNA DSBs, as measured by 53BP1 and γH2AX colocalization (Figure 3F and 3G, Figure S2).

Mice with an S1932P mutation in *Polq* (the “*chaos1*” allele) have an increased spontaneous frequency of micronuclei [13]. We generated a human *POLQ* cDNA mimicking the *chaos1* mutation (S1977P), but attempted expression of POLQ with this mutation in 293T cells did not yield detectable protein (Figure S3). This suggests that the *chaos1*-encoded mutant protein is unstable, consistent with the finding that *chaos1* mice have a phenotype essentially indistinguishable from *Polq* knockout mice [13].

### POLQ operates in a pathway of altEJ during mouse Ig class-switching

Immunoglobulin class-switch recombination (CSR) uses DNA end joining to exchange one constant region of an antibody gene for another constant region. CSR can occur by both Ku-dependent classical non-homologous end joining and Ku-independent altEJ [20]. The overall frequencies of CSR are similar in *Polq*-defective mice [29] and cultured B cells [30]. To determine whether POLQ is involved in a mechanistically distinct subset of CSR joins, we isolated and analyzed DNA sequences at such joins. Naïve B cells were isolated from the spleens of wild-type and *Polq*-null mice and stimulated for IgM to IgG class switching, and then the fraction of IgG1-positive B cells was measured by flow cytometry. Parallel B-cell cultures were incubated with NU7026, a DNA-PKcs inhibitor that suppresses cNHEJ [31]. It has been shown that B cells incubated with NU7026 have an increased proportion of CSR junctions with >1 bp insertion at the junction [31]. This suggests that when a pathway of altEJ is used during CSR, it more frequently results in insertion of nucleotides.

**Figure 4:**
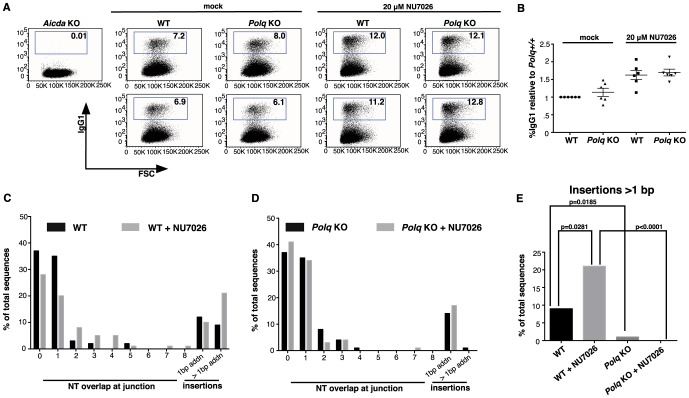
Insertions >1 nt at CSR junctions are *Polq-*dependent. Isolated wild-type (WT) and *Polq^−/−^* (KO) naïve splenic B cells were stimulated for CSR and either mock-treated or treated with NU7026. (A) Cells were assayed for IgG1 levels (y-axis) by flow cytometry. The x-axis sort is on forward scatter (FSC) for cell viability. *Aicda^−/−^* splenic B cells were used as a negative control. Numbers in boxes show the percentage of the population that is IgG1 positive; in (B) these data are plotted relative to wild-type. Genomic DNA isolated from B cells of wild-type (C) and *Polq* KO (D) mice was amplified by PCR and 100 Sμ-Sγ1 junctions from each group were sequenced and analyzed for overlaps and insertions at breakpoints. (E) Insertions >1 nt are plotted; p-values were determined by a two-tailed Fisher's exact test. Cell viabilities were comparable between genotypes.

We found that B cells from *Polq*-proficient and deficient mice had similar overall frequencies of CSR (Figure 4A), and inhibition of DNA-PKcs increased the frequency of CSR in both genotypes by 1.5 to 2 fold (Figure 4B). The Sμ-Sγ1 junction was then sequenced from 100 clones of each group of IgG1-positive B cells. These data revealed that in wild-type B cells, insertions of >1 bp at Sμ-Sγ1 junctions, that are thought to be altEJ-dependent, comprised about 9% of total events, and that this increased to ∼21% in cells incubated with NU7026 (Figure 4C, Table 1).

**Table 1:** Sequence composition of >1 nucleotide CSR insertions.

	Sμ (J00440)	Insertion	Sγ (D78344)		Homology
	***Polq* WT**				
4648:4677	GGGACCCAGGCTttGAAGGCAATCCTGGGA	GCACTTC	GTACTTATAGAGGAACAGGGGCAGcgTAGA	2888:2917	
5161:5190	ACTGTAATGAACTGGAATGAGCTGGGCCGC	CCT	GGAAATCAGGACAGGTACAAGcGTGTGGAT	7925:7954	
5291:5320	TTCTGAGCTGAGATGAGCTGGaGTGAGCTC	TTACC	GGTAGCTATAGGGAATACAGAACAGGTGGG	2653:2682	
4974:5003	GAAGGCgAGACTCATAAAGCTTGCTGAGCA	**CCAGGACA**	CCCTGTAGGGTAGCTGTAGGGAAATCAGGA	7906:7935	Sγ2278:2285
5206:5235	TGGCTTAACCGAGATGAGCCAAACTGGAAT	CCC	GAATAGCTACAGGGGAGCCAGGAGAGGTAG	2753:2782	
4891:4920	AATTGAGAAAGAATAGAGACCTGCAGTTGA	TC	CTGATGGCAAATGGAAGGGCAGAGACCCAG	2957:2986	
5110:5139	AGGTGATTACTCTGAGGTAAGCAAAGCTGG	CA	CCAGGTCTGCAGCTACATACGGGTAAGCAG	2694:2723	
5297:5326	GCTGAGATGAGCTGGGGTGAGCTCAGCTAT	TT	TATAGGGAGCCAGGACAGGTGGAAGTGTGG	7602:7631	
4667:4696	CAATCggGGGATTCTGGAAGAAAAGATGTT	CC	GGATCAAGGCAGAACAGGTCCAGGGGTGCC	2840:2869	
	***Polq* WT+NU7026**				
5432:5461	TAGGGTGAGCTGAGCTGGGTGAGCTGAGCT	CCCAGT	AGTGTGGGGATCCAGGTAAGGCTGGACTGG	8264:8293	
4620:4649	TGGAAGCTAATTTAGAATCAAGTAAGGAGG	**CAAGACAGGTGG—GTGTGGGGATCAAGG**	AGGAGAAATGGAAGAATGCAGATTCCAAAC	8030:8059	Sγ2820:2848
4907:4936	AGACCTGCAGTTGAGGCCAGCAGGTCGGCT	ACTA	TGGAACTGTGGGGACCCTGTAGGGTAGCTG	7892:7921	
4787:4816	GTTGTTAAAGAATGGTATCAAAGGACAGTG	**CTAgAAcGTAcT**	TCAAGGCAGAACAGGTCCAGGGGTGCCAGG	2843:2872	Sμ3545:3556
5231:5260	GGAATGAACTTCATTAATCTAGGTTGAATA	**GGTGGAAGTGTGGGG**	AGGGCAGCCAGGACAGGTGGAAATGTGGTG	7750:7779	Sγ4882:4896
4879:4908	ACAGCTGTACAGAATTGAGAAAGAATAGAG	**TGGGG—AGCTCAGCTATGCTAC-CGTGTTG-GG**	CAGGAAGAATAGCTACAGGGGAGCCAGGAG	2747:2776	Sμ5309:5342
5291:5320	TTCTGAGCTGAGATGAGCTGGaGTGAGCTC	TTACC	GGTAGCTATAGGGAATACAGAACAGGTGGG	2653:2682	
4974:5003	GAAGGCgAGACTCATAAAGCTTGCTGAGCA	CTACGACA	CCCTGTAGGGTAGCT GTAGGGAAATCAGGA	7906:7935	
5237:5266	AACTTCATTAATCTAGGTTGAATAGAGCTA	**GAC—GTaGCATTGTGTGacTC**	CAGGCACAGTAGCTATAGGGGaGCCAAGAC	2796:2825	Sγ2575:2596
4989:5018	AAAGCTTGCTGAGCAAAATTAAGGGAACAA	TTGAG	TGAGGCAGGTAAGAGTGTGGGAACCCAGTC	8429:8458	
5245:5274	TAATCTAGGTTGAATAGAGCTAAACTCTAC	**AGTGCAGA**	TGAGGCAGGTAAGAGTGTGGGAACCCAGTC	8429:8458	Sγ8821:8828
4685:4714	AGAAAAGATGTTTTTAGTTTTTATAGAAAA	GTAAT	TATAGGGGaGCCAAGAaAGGTGGAAGTGTG	2809:2838	
4796:4825	GAATGGTATCAAAGGACAGTGCTTAGATCC	G**AGGTGAGTGTGAGAGGACA** AA	AAGTTtAGTAgTTATAGAGGAACAGGGGCA	2881:2910	Sμ4827:4845
5088:5117	GCTTCTAAcATGCGCTAAACTGAGGTGATT	CCAAG**GACCCAGGCAGAGCAGCTCCAG** TAGGCCA	CAAtcACAAGGGAACTGATGGCAAATGGAA	2943:2972	Sγ4014:4035
4663:4692	AAGGCAATCtTGGGATTCTGGAAGAAAAGA	CCAT	GAAGTGTAGTGACCCttGAAGAATAGCTAC	2733:2762	
5002:5031	CAAAATTAAGGGAACAAGGTTGAGAGCCCT	TC	GGAATTGTGGTGACCCAGACAAAACAGCTA	7817:7846	
4684:4713	AAGAAAAGATGTTTTTAGTTTTTATAGAAA	CC	ACAGGGAAGCTATAttAAAACCAGGACAcG	8132:8161	
5133:5162	AAGCTGGGCTTGAGCCAAAATGAAGTAGAC	GC	TGGAAGGGCAGAGACCCAGACTAAATGGCT	2968:2997	
5210:5239	TTAACCGAGATGAGCCAAACTGGAATGAAC	TA	AAAACCAGGACAGGAGGAAGAATGGGGATC	8148:8177	
4816:4845	GCTTAGATCCgAGGTGAGTGTGAGAGGACA	AA	AAGTTtAGTAgTTATAGAGGAACAGGGGCA	2881:2910	
4897:4926	GAAAGAATAGAGACCTGCAGTTGAGGCCAG	TT	AGGGTGTGGATCCAGGCAGGGTAGCTATAG	2634:2663	
	***Polq* KO**				
5360:5389	AGCTACTCTGGAGTAGCTGAGATGGGGTGA	TT	CAAATGGAAGGGCAGAGACCCAGACTAAAT	2964:2993	

30 bases flanking each side of the insertion are shown. The numbers separated by colons give the position relative to the beginning of the Sμ and Sγ genomic sequences. New mutations (different from the reference sequence) are shown in lower case. For some longer insertions (indicated in bold), homologies were identified in the switch region, at the positions indicated in the right column. Microhomologies at the junction site are underlined. A dash (-) indicates a base deletion. Sequences (graphed in Figure 4E) are from *Polq* ^+/+^ (WT) and *Polq* ^−/−^ (KO) splenic B cells that were treated with NU7026 or mock-treated.

Strikingly, in cells lacking *Polq*, this class of insertions at CSR junctions was absent, even in the presence of NU7026 (Figure 4D, only one insertion of 2 bp observed). Insertion of >1 bp therefore requires POLQ. This class of *Polq*-dependent joining events included insertions of between 2 and 35 bp. For longer insertions (greater than ∼10 bp) homologous sequences were unambiguously detected up to 2–5 kbp away from the junction site (Table 1), as has been reported for long insertions at Sμ-Sγ1 junctions in ATM-defective B cells [31]. This suggests that most or all of such insertions are formed in a templated manner during altEJ by POLQ.

### Loss of *Polq* impairs an altEJ pathway but not cNHEJ in cultured cells

The most important factor in determining which double-strand break repair pathway is used is whether or not the 5′ termini of broken ends are resected [32]. Ends with little or no single stranded overhang are typically rejoined by Ku-dependent cNHEJ. In contrast, CtIP and MRN-dependent resection of 5′ termini generates ends with extended single stranded 3′ overhangs; resection is thought to block cNHEJ [33] and enable repair by altEJ [34], [35].

**Figure 5:**
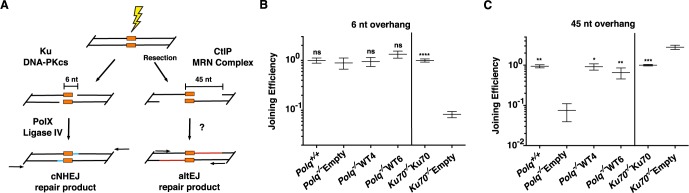
End joining with extrachromosomal substrates. (A) Substrates were designed to resemble DNA double-strand breaks that are repaired through Ku-dependent NHEJ (6 nt tail with 4 nt of terminal complementary sequence) or alternate end-joining of resected DNA substrates (45 nt tail with 4 nt terminal complementary sequence), introduced into cells, and joining of head-to-tail products assessed by qPCR. (B) qPCR for the classical NHEJ assay uses primers to detect all events having sequences in the duplex immediately flanking the break. Joining efficiencies are expressed as fractions of the mean joining determined for matched wild controls (*Polq* ^+/+^ or Ku70 complemented lines, as appropriate). Three independent triplicate measurements were made for the *Polq* cell lines and two independent triplicates for the *Ku* cell lines. Error bars represent the standard error of the mean. Joining efficiency was not significantly different, whether cells were deficient in *Polq* (*Polq* ^−/−^Empty) or not (*Polq* ^+/+^, *Polq* ^−/−^WT4, *Polq* ^−/−^WT6), but was different when cells expressed Ku (*Ku70* ^−/−^Ku70) when compared to *Ku70* ^−/−^Empty cells (t-test, p<0.05) (C) qPCR for the altEJ assay used primers to detect that subset of products including at least 10 nt of each 3′ overhang. Mean relative joining efficiencies, standard error of the mean, and statistical analysis performed as for panel B. Joining efficiency was significantly different in cells expressing *Polq* (*Polq* ^+/+^, *Polq* ^−/−^WT4, or *Polq* ^−/−^WT6) when compared to *Polq* ^−/−^Empty cells (p<0.05), and in cells expressing Ku (*Ku70* ^−/−^Ku70) when compared to *Ku70* ^−/−^Empty cells (t-test, p<0.05). The background observed in a mock transfected sample was determined to be 0.038, +/− 0.02 of wild-type controls. p values are represented as: * p<.05, ** p<.01, *** p<.001, ****p<.0001.

To analyze differing requirements for end joining, with or without end resection, we generated two linear DNA substrates with 3′ single stranded overhangs; one with a short overhang (6 nt), and one a long overhang (45 nt, a “pre-resected end”) (Figure 5A). Both can be aligned with the same 4 nt of terminal complementary sequence. These substrates were then introduced into wild-type mouse fibroblasts or fibroblasts harboring deficiencies in *Ku70* or *Polq*. Repaired products were recovered from cells and quantified. Repair of the short overhang substrate was, as anticipated, over 10-fold less efficient in cells without Ku70 (Figure 5B) when compared to Ku70-complemented controls. The absence of *Polq^−/−^* had no consequence for repair of this substrate.

End joining with the 45 nt overhang substrate was assessed using qPCR primers located to ensure that at least 10 nt of overhang was included in joined products (Figure 5A). Recovery of these products was no longer dependent on Ku; instead, it was increased 2.8-fold in *Ku70*-deficient cells (Figure 5C). This is consistent with previous studies arguing Ku suppresses repair by altEJ. Strikingly, joining of the long overhang substrate in *Polq^−/−^* cells was reduced 10-fold, near background levels of signal observed using this assay. Complementation of the knockout cells with POLQ returned joining to wild-type levels (Figure 5C). These data demonstrate that POLQ participates in some form of alEJ, but cells lacking POLQ maintain proficiency for cNHEJ.

### POLQ extends 3′ DNA ends in a template-dependent manner

**Figure 6:**
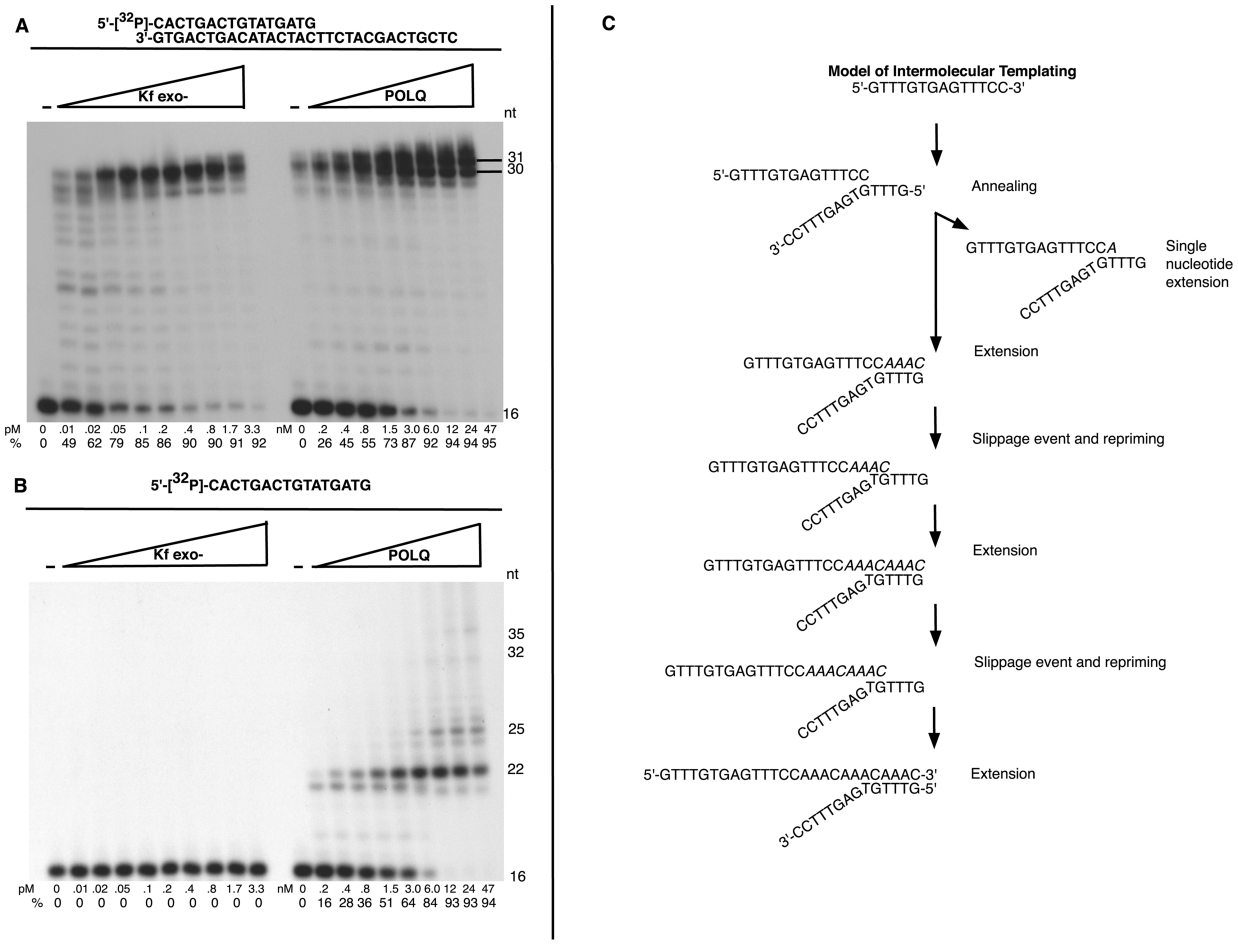
Unique template dependent DNA polymerase activity of POLQ. Exonuclease-defective *E. coli* pol I Klenow fragment (Kf exo-) or POLQ was incubated at the indicated protein concentrations with (A) a 5′-^32^P-labeled primer 16-mer and 30-mer complementary template, (B) 5′-^32^P-labeled 16-mer primer and no template. All reaction mixtures included all four deoxynucleotide triphosphates and were incubated at 37°C for 10 min (A) or 20 min (B). The first lane contained no enzyme. The percentage (%) of the primer extended is shown below each lane. (C) Model of intermolecular templating performed by POLQ in the process of extending a different single-stranded oligonucleotide, used to produce the data in Table S1. This model depicts a 12 nt extension product in Table S1. The product can be produced by a series of annealing, extension, slippage and repriming events.

Our results demonstrate that POLQ is necessary to form the insertions found in CSR junctions in a process of altEJ. We next sought to determine the mechanism. Like other DNA polymerases, an active polymerase fragment of POLQ [36] can catalyze template-dependent DNA synthesis from an annealed primer (Figure 6A). As is common for family-A DNA polymerases, only a single nucleotide is added to the end of duplex DNA [5]. Unusually, however, POLQ can catalyze extension of single-stranded oligonucleotides [37]. It was unclear whether this reflects a robust terminal deoxynucleotide transferase activity of POLQ on single-stranded DNA, or some form of template-dependent synthesis. For example, POLQ can extend a single-stranded 16-mer oligonucleotide provided without a complementary template (products up to 35 nt long), while *E. coli* pol I Klenow fragment has no activity on this substrate (Figure 6B). The major 22 nt extension product produced by POLQ on the 16-mer used in Figure 6B may be accounted for by inter- or intra-oligonucleotide pairing (Figure S4C). Neither POLQ nor Klenow fragment could extend an oligonucleotide that was incapable of annealing to itself (Figure S4) [37].

To identify the mechanism of 3′ single-stranded DNA extension by POLQ, we used a different single-stranded oligonucleotide designed to be unable to form self-complementary base pairs longer than a single nucleotide [37], and sequenced the products of POLQ-mediated extension. Individual extension products of 1 to 30 nt were found (Table S1). Most of the sequenced extension products feature AAC or AAAC sequences that could arise from copying GTTT sequences in the template via inter- or intra-molecular priming and re-priming (Figure 6C) following minimal base pairing at the 3′-primer end. These data reveal that POLQ uniquely extends 3′ DNA tails through template-dependent DNA synthesis from a primer with minimal base pairing and that the polymerase lacks true TdT-like activity. POLQ indeed has unique biochemical properties compatible with these observations. Unlike other DNA polymerases, POLQ can efficiently extend a DNA chain with a nucleotide incorporated opposite an abasic site [5], or from a mismatched primer-terminus [38]. Further, there is evidence that primers slip on DNA templates with an increased frequency during POLQ-mediated synthesis, as shown by the high frequency of single base pair frameshift mutations generated by purified POLQ [39].

### POLQ suppresses chromosomal translocations in B cells

**Figure 7:**
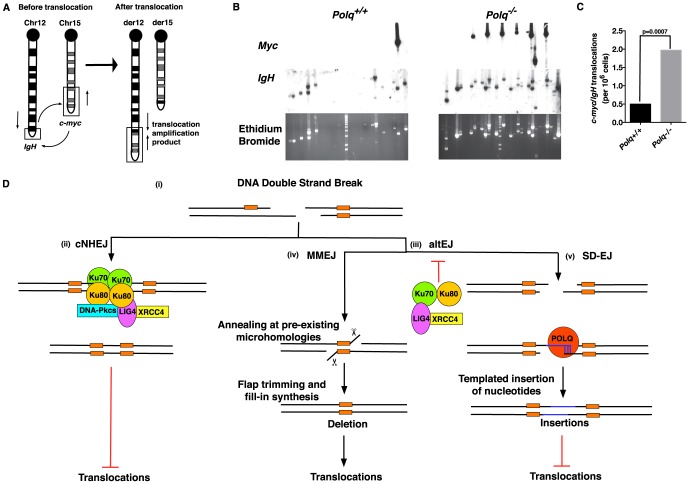
POLQ suppresses chromosomal translocation *in vivo*. (A) Representative schematic for the *Myc*/*IgH* translocation assay. PCR amplification primers are represented by black arrows. Closed circles denote centromeric locations on the chromosomes. Naïve B cells from wild-type (WT) or *Polq^−/−^* mice were assayed for translocations after 72 hr in culture. (B) Representative agarose gels stained with ethidium bromide and Southern blots with *IgH* and *Myc* probes. Each lane contains the DNA content of 1×10^5^ genomes. Three independent experiments were performed. (C) Frequency of translocations was plotted and p-values determined using two-tailed Fisher's exact test. Frequencies were calculated from total translocations (*Polq^+/+^*: 5; *Polq^−/−^*: 17) divided by total number of genomes surveyed (9.6×10^6^). (D) Model of end joining-mediated repair of DNA double-strand breaks (DSBs). (i) Schematic representing a DSB with existing microhomologies shown in orange. (ii) DSBs are preferentially processed by classical non-homologous end joining (cNHEJ), dependent upon Ku70–80 and Ligase4-XRCC4. This pathway is not thought to promote DNA translocations. In the absence or impairment of critical cNHEJ factors (iii) alternative end joining (altEJ) pathways are utilized. These pathways appear to be suppressed by Ku70–80 and Ligase4-XRCC4. The MMEJ pathway (iv) can orchestrate annealing of ends at pre-existing microhomologies (2–5 bp) resulting in a net deletion of genomic information. Utilization of this pathway can enhance the formation of chromosomal translocations. In the SD-EJ pathway (v) POLQ can catalyze extension of minimally paired 3′ single-stranded DNA ends (shown in blue) to facilitate end joining and suppress the formation of chromosomal translocations.

Double-strand breaks initiated by AID activity in the immunoglobulin heavy chain (IgH) locus of B cells are necessary to generate immunological diversity, but breaks are sometimes generated at other chromosomal sites, providing an opportunity for dangerous chromosome translocations [21], [22], [40], [41]. For instance the oncogenic *Myc/IgH* translocation that causes Burkitt lymphoma is AID-dependent and requires breaks at both loci, with breaks in the *Myc* gene rate-limiting [42]. An altEJ process is implicated in the formation of oncogenic translocations in lymphoid tissues, including the *Myc*/*IgH* translocation in murine B cells [21], [43], [44]. cNHEJ suppresses the formation of such chromosomal translocations [45]. To determine the role of POLQ in chromosomal translocations, *Polq^+/+^ and Polq^−/−^* naïve splenic B cells were stimulated in culture and assayed for the frequency of *Myc/IgH* translocations (Figure 7A). Notably, in the absence of *Polq* there was a 4-fold increase in translocation frequency (Figure 7B and C). This indicates that mammalian POLQ acts in a subset of altEJ events to suppress chromosomal translocations. Additionally, an increase in intramolecular *IgH* rearrangements was found in B cells lacking *Polq* (Figure 7B). Therefore, although POLQ is involved in an altEJ pathway, it prevents rather than promotes chromosomal instability, rearrangements and the formation of *Myc*/*IgH* translocations.

## Discussion

### POLQ suppresses hypersensitivity to direct DNA double-strand breaks

We show that in mammalian cells, POLQ has a specific role in defense against DNA damaging agents that cause direct DNA double-strand breaks, including ionizing radiation, bleomycin, and topoisomerase inhibitors. Our findings indicate that POLQ participates in a novel pathway of alternative-end joining of DSBs, a process that can occur throughout the cell cycle in mammalian cells [17]. The minimal additional sensitization to camptothecin by olaparib in *Polq*-defective cells suggests that one function of PARP is to participate in a *Polq*-dependent altEJ pathway. Our experiments indicate that POLQ is an important factor in DNA DSB repair in all cells, not just cells of the hematopoietic lineage. Indeed, *Polq* is broadly expressed in murine tissues (Figure S5).

Mutants of POLQ homologs in *Arabidopsis* (TEBICHI), *C. elegans* (polq-1), and *Drosophila* (Mus308) are hypersensitive to ICL-inducing agents [3], whereas *Polq*-defective mammalian cells are not appreciably hypersensitive to such agents (Figure 1). This difference may arise because of differences between organisms in the priority of DNA repair pathway engagement. In proliferating mammalian cells, ICLs are usually dealt with through the Fanconi anemia pathway, which produces enzymatically induced double-strand breaks that are channeled into homologous recombination repair [46]. In *Drosophila* and some other organisms, an altEJ-dependent pathway may be more important for resolving ICL-associated double-strand breaks. Although *Drosophila Mus308* mutants are not hypersensitive to IR, pronounced IR sensitivity occurs in a double mutant when HR is also inactivated [47]. The phenotypic consequences of POLQ-dependent altEJ of double-strand breaks may thus depend on the relative dominance of HR which varies between organisms.

We show here that the DNA polymerase activity of POLQ is necessary to prevent cell death and chromosome breaks (micronuclei) caused by a double-strand break-inducing agent. Disruption of the ATPase activity in the helicase-like domain of POLQ did not, however, alter the correcting function of POLQ addition to knockout cells. A previous study with mouse cell lines suggested that disruption of the polymerase domain of the murine *Polq* gene is less severe than complete disruption of *Polq* [30], but the result is difficult to evaluate in the absence of quantitative measurements of expression of the partially deleted form. No activity has yet been shown for the helicase-like domain, other than DNA-dependent ATPase function [4]. It is likely that an additional role remains to be discovered that is dependent on the ATPase function of POLQ.

### POLQ aids DNA double-strand break repair through alternative end joining and nucleotide insertions

When double-strand breaks form in mammalian cells, a majority will be repaired through cNHEJ. However, a subset of these breaks will be handled by alternative end-joining pathways in situations where the DNA end is not compatible with processing by Ku-dependent cNHEJ, or if core components of the cNHEJ machinery are absent or unavailable (Figure 7D). In general, altEJ is defined as a means for repair of chromosome breaks that is exclusive of Ku-dependent, classically defined NHEJ [48], and dependent on factors (CtIP, MRN) that resect double-strand breaks to generate extended 3′ ssDNA tails [34], [35] (Figure 5A). Accordingly, we showed joining of a “pre-resected” extrachromosomal substrate (substrate with 45 nucleotide 3′-ssDNA tails) was stimulated in *Ku*-deficient cells, similar to results using chromosomal substrates [35]. Joining of this substrate was also dependent on *Polq* (Figure 5C). Our experiments thus define an altEJ subpathway in mammalian cells that involves POLQ (termed synthesis-dependent end joining, SD-EJ, in Figure 7D), Additional Polq-independent altEJ subpathways may also be operational (Figure 7D). To some extent, different end-joining pathways can be been distinguished from one another by the ligase employed in the pathway, with DNA ligase IV (LIG4) suggested as essential for cNHEJ, and DNA ligase III (LIG3) for altEJ in mammalian cells [21], [43], [49]. There are caveats, however. For example, some functional redundancy is apparent between LIG1 and LIG3 in altEJ [44], [50]–[52]. Ligase deficiencies may thus not be the best marker for distinguishing different end-joining pathways. For the altEJ subpathway under consideration here, dependence on POLQ is the best available definition.

The biochemical properties of POLQ provide a mechanistic explanation for its contribution to altEJ. POLQ has a unique ability to add nucleotides to the 3′ ends of single-stranded DNA [37], primed by minimal pairing with other available DNA molecules (Figure 6 and Figure S6). Synthesis by POLQ in this context is consistent with the unusually efficient ability of the polymerase to extend from mismatched DNA termini [5], [38], and its tendency towards primer-template slippage [39]. In further biochemical experiments it will be of interest to examine the action of POLQ and DNA ligases at double strand breaks with 3′-single-stranded overhangs that closely mimic the resected ends of a DNA double-strand break. In vivo studies with such substrates, including those that can form hairpins in the single-stranded region, would give insight as to the preferred structures for POLQ-catalyzed extension.

Unique to the POLQ-dependent altEJ process are frequent joins displaying templated DNA insertions. Some form of altEJ has been implicated in resolution of a subset of double-strand break intermediates in CSR, producing templated insertions [20]. Our data support a role for POLQ in generating the CSR products with these templated insertions. These events are consistent with the templated insertions that occur during Mus308-dependent repair of directed double-strand breaks in *Drosophila* [47], [53] and in *C. elegans* [54]. In the absence of POLQ, the lack of insertion-containing joins is observed, but the global CSR frequency is relatively unchanged (Figure 4). These insertions are best explained by repeated initiation of synthesis by POLQ (Figure 6C) on template sites, ultimately leading to a joined product.

### POLQ prevents the formation of *Myc/IgH* chromosomal translocations

In the absence of POLQ, we found a ∼4-fold increase in the formation of the oncogenic translocation *Myc*/*IgH* in mice. This increase is comparable to that seen in B cells that have lost *Tdrd3*, a regulator of R-loop formation during transcription [55] and *miRNA-155* which regulates AID and suppresses oncogenic translocations [56]. In the absence of *Polq* there is also an apparent enhancement of rearrangement events in the *IgH* locus, consistent with the elevated level of chromosomal instability observed in cells lacking POLQ [57].

altEJ is typically associated with frequent annealing of the DNA ends at existing microhomologies (2–5 bp) and large deletions at repair junctions [19]. Since translocations commonly feature such microhomologies at their breakpoint junctions [58], [59] and occur more frequently in cNHEJ defective cells, altEJ is considered the primary mechanism by which translocations occur. Thus, a striking finding of the present work is that the formation of *Myc/IgH* translocations is suppressed when the POLQ-dependent altEJ subpathway is operational. It is possible that DNA DSBs persist for a longer time in the absence of POLQ, giving more opportunity for the formation of translocations. Alternatively, the POLQ-dependent pathway may be the most efficient at repairing a structurally distinct class of translocation-prone DNA breaks.

These studies clearly define a role for POLQ in the repair of DNA strand-breaking agents and provide a mechanism of template-dependent extension of DNA ends necessary to repair breaks in a subpathway of altEJ. This distinct altEJ pathway is necessary to prevent the formation of chromosomal translocations as shown by our *in vivo* experiments. It has been suggested that suppression of POLQ may be useful in increasing the efficacy of DNA damaging treatments in cancer [3], [23], [60]. This promising prospect should be tempered with the knowledge that loss of POLQ may also lead surviving cells to be prone to potentially oncogenic chromosome translocations.

## Materials and Methods

### Ethics statement

Research mice were handled according to MD Anderson Cancer Center Institutional Animal Care and Use Committee policies and protocol 08-08-08732. Mice were euthanized by CO_2_ euthanasia followed by cervical dislocation.

### Cellular proliferation assay

*Polq^+/+^* and *Polq^−/−^* bone marrow stromal cells and mouse embryonic fibroblasts were plated in triplicate (200,000 cells per 10 cm dish) with 15 mL of complete media (Dulbecco's Modified Eagle Medium+Glutamax, 10% FBS, 1% PennStrep). On the indicated days, cells were trypsinized and live cells were counted using trypan blue exclusion (Countess automated cell counter, Life Technologies). Experiments were repeated three times in order to generate standard deviations. Viability was consistently high for all cell lines examined (>95% trypan blue-excluding cells).

### Clonogenic assays with bone marrow stromal cells

For X-irradiation 5×10^5^ cells were plated on a 10 cm plastic culture dish, and exposed the following day at 2 Gy/min, 160 kV peak energy (Rad Source 2000 irradiator, Suwanee, GA). Cells were then trypsinized for replating. For UVC-irradiation (254 nm peak germicidal lamp) cells were irradiated in 500 µl PBSA (10^5^ cells/ml) at 5 J m^−2^ min^−1^ and then plated. For psoralen-UVA treatment, 5×10^5^ cells were plated on a 10 cm dish and incubated in medium with the indicated concentration of HMT-psoralen for 1 h, the dish was irradiated for with 0.9 kJ m^−2^ UVA (365 nm peak, 30 min, 0.5 mJ m^−2^ sec^−1^), the psoralen-containing medium was removed, and the dish UVA-irradiated in fresh medium for a further 30 min before replating. Chemicals were added at the indicated concentrations to dishes at the beginning of the experiment. Drugs were solubilized in ethanol (mitomycin c), DMSO (ICRF-193, etoposide, camptothecin, HMT-psoralen, temozolomide, olaparib), or 150 mM NaCl (cisplatin). All chemicals were from Sigma (St. Louis, MO) except ICRF-193 (Enzo LifeScience, Farmingdale, NY), olaparib (AZD2281, Selleck Chemicals, Houston, TX), and mitomycin c (Calbiochem, Darmstadt, Germany). Cells were plated in triplicate in 10 cm dishes and grown for 7–10 days before being fixed and stained with crystal violet. Colonies of 50 or more cells were quantified and experiments were repeated three times to generate standard deviations. A clonogenic assay was performed with *Rad51D^+/+^* and *Rad51D^−/−^* Chinese hamster ovary (CHO) cell lines exposed to varying concentrations of olaparib.

### Micronucleus assay

BMSCs were plated at 1.5×10^4^ cells per well in chambered slides and treated with the indicated amount of x-rays or etoposide the following day. 48 hr later, cells were fixed with 2% para-formaldehyde, stained with DAPI and coverslipped. Micronuclei were scored by immunofluorescence for 300 cells per group. Experiments were repeated three times to generate standard deviations.

### Human cell transfections

293T cells (kindly provided by Dr. Christopher Bakkenist, University of Pittsburgh Medical School) were plated at 150,000 cells in six-well plates and transfected the following day with 2.5 µg of either pCDH (System Biosciences, Mountain View, CA) containing empty control, POLQ, POLQ-K121M, POLQ-D2330A,Y2331A, POLQ-S1977P, or POLQ-DM cDNA using Lipofectamine 2000 (Life Technologies) according to manufacturer's specifications. 48 hr after transfection, cells were harvested for RNA isolation (RNeasy, Valencia, CA) or immunoblotting.

### Immunoblotting

For immunoblots, cells transfected in six-well dishes were resuspended in 200 µL of 2× SDS loading buffer (4% SDS, 0.2% bromophenol blue, 20% glycerol, 100 mM Tris HCl pH 6.8, 12% 2-mercaptoethanol) and heated at 95°C for 5 min. 20 µL of extract was separated on a 4–20% polyacrylamide gel, transferred to PVDF membrane, blocked, and blotted with anti-alpha-Tubulin (Abcam, Cambridge, UK) ab4074, 1∶10,000), anti-FLAG (Sigma F7425, 1∶5,000), anti-PCNA (Santa Cruz, Santa Cruz, CA, sc-56, 1∶1,000), anti-HA (RW, 1∶10,000), or anti-POLQ (MDACC POLQ20, 1∶250) antibodies and corresponding secondary antibodies (Sigma A0168, A0545; 1∶10,000) and visualized with ECL reagent (Pierce, Rockford, IL).

### Generation and complementation of *Polq* MEFs

*Polq*-null (*Polq^−/−^*) mice [13] were obtained from Jackson Laboratories and maintained on a C57BL/6J background. Isogenic primary MEFs were generated from 13.5 day pregnant females and cultured in a 2% O_2_ atmosphere. MEFs were then transfected with 1 µg of pSV-Tag [61], [62] and grown in atmospheric oxygen for six population doublings to allow for immortalization. To generate lentivirus used for transduction, 293T cells were cotransfected with psPAX2 (6 µg), pMD2G (6 µg), and pCDH (12 µg) expression vector (See Text S1 for construction of expression vectors) using Lipofectamine 2000. One day prior to transduction *Polq^−/−^* MEFs were seeded into a 10 cm dish at 1.5×10^5^ cells with 12 mL complete media. 48 hr post-transfection virus-containing media was harvested, filtered through a 0.45 µm syringe filter and used to replace the media on the plated MEFs. MEFs were incubated in the virus-containing media for 24 hr before being split into T-75 flasks and allowed to grow to 80% confluence before undergoing three weeks of puromycin selection (2.5 µg/mL). Following selection, pure clones were isolated and cultured with complete media containing puromycin (1 µg/mL).

### Quantitative real-time PCR analysis of complemented MEFs

RNA isolated from the complemented MEF lines were analyzed for quality and purity using RNA 6000 Nano kit (Agilent Technologies, Santa Clara, CA). 1 µg of total RNA was used to generate cDNA using the High Capacity cDNA RT kit (Life Technologies). qPCR analysis was performed in triplicate using the ABI Prism 7900 HT thermocycler and the following Taqman Probe set or primer set with iTAQ SYBR Green Supermix with ROX (Bio-Rad, Hercules, CA): MmPolQ_FWD 5′-GGCTCTGAAGAACTCTTTGCCTTT-3′, MmPolQ_REV 5′-GCTGCTTCCTCTTCTTCATCCA-3′, probe 5′-TCCGGGCACTTTTG-3′; HsPOLD1_FWD 5′-CGACCTTCCGTACCTCATCTCT-3′ HsPOLD1_R 5′-ACACGGCCCAGGAAAGG-3′, probe 5′-CCCTCAAGGTACAAACAT-3′; Qexon FWD 5′-TGCCTTTCAAAAGTGCCCGGAAGGC3′, Qexon REV 5′-TGCCAGTCACCCANATAGTTCNCAT-3′. Data were analyzed using the ΔΔCt method. For absolute quantification, titration of pCR-XL-TOPO/MmPolQ and pET/MmPold1 plasmids were used to generate standard curves for expression. Transcript abundance was determined by extrapolation from linear regression analysis of best fit lines from titration experiments. GAPDH was used as an internal control in all experiments.

### Bleomycin sensitivity

Complemented MEF lines were plated in triplicate into white 96 well plates at 1250 cells per well and grown overnight using complete media containing puromycin (1 µg/mL). The following day, cells are cultured with complete media containing the indicated amounts of bleomycin (dissolved in 150 mM NaCl) for 24 hr before the media was replaced. Cells were allowed to recover for 72 hr before cellular viability was measured using the ATPlite 1Step kit (Perkin Elmer, Waltham, MA) using a Biotek plate reader. Experiments were repeated three times.

### Immunofluorescence

Complemented MEF lines were plated at a density of 1.5×10^4^ cells per well in 4-well chamber slides and the following day were irradiated with either 0 or 6 Gy of x-rays. Media was changed and cells were allowed to recover for 48 hr after damage before fixation with 2% para-formaldehyde and permeabilized with Triton X-100. Samples were blocked with donkey serum for 30 minutes before being incubated overnight with primary antibodies against 53BP1 (Bethyl, Montgomery, TX, A300-272A, 1∶500) and γH2AX (EMD Millipore 05-636, 1∶400). Cells were later incubated with AlexaFluor-488 goat-anti-mouse or AlexaFluor-594 goat-anti-rabbit secondary (Life Technologies, 1∶1000) and then stained with DAPI before being coverslipped. Cells were scored for DSBs by enumerating the percentage of cells with >2 53BP1 foci and >2 γH2AX foci [61], [63]. The majority of cells that contained >2 foci for each of the DSB markers, exhibited colocalization of the foci. Cells with pan-staining of γH2AX were not included in the analysis as they are proposed to represent pre-apoptotic cells [64]. Many of the cells with 53BP1 foci, exhibited enlarged foci that are associated with nuclear OPT (Oct-1, PTF, transcription) domains that sequester damaged DNA in G1 [65], [66]. Thus, most of the MEFs that were foci positive contained DSBs [65]. DAPI-stained micronuclei were also scored. For each experiment 250 cells were scored for three independent experiments for a total of 750 cells.

### DNA polymerase assays

POLQ was purified as described [36]. Klenow Fragment (3′→5′ exo-) was purchased from NEB. POLQ was diluted in buffer containing 30 mM Tris-HCl pH 8.0, 50 mM NaCl, 2 mM DTT, 10% glycerol, 0.01% Triton X-100, and 0.1% BSA. Klenow Fragment (3′→5′ exo-) was diluted in buffer containing 25 mM Tris-HCl pH 7.4, 1 mM DTT, and 0.1 mM EDTA. POLQ reaction mixtures (10 µl) contained 20 mM Tris-HCl pH 8.8, 4% glycerol, 2 mM dithiothreitol (DTT), 80 µg/ml bovine serum albumin (BSA), 8 mM MgCl_2_, 0.1 mM EDTA, 100 µM of each dNTP, 30 nM of the primer-template or primer (see Text S1). Klenow Fragment (3′→5′ exo-) reaction mixtures (10 µl) contained 10 mM Tris-HCl pH 7.9, 50 mM NaCl, 1 mM DTT, 10 mM MgCl_2_, 100 µM of each dNTP, and 30 nM of the primer-template or primer. After incubation at 37°C for 10 min for a 16-1+PA42 substrate or 20 min for 16-1, C20, C19THF substrates, reactions were terminated by adding 10 µl of formamide stop buffer (98% formamide, 10 mM EDTA pH 8.0, 0.025% xylene cyanol FF, 0.025% bromophenol blue) and boiling at 95°C for 3 min. Products were electrophoresed on a denaturing 20% polyacrylamide-7 M urea gel, exposed to BioMax MS film, and analyzed with a STORM 860 Phosphor Imager (Molecular Dynamics).

### Extrachromosomal substrate assays

A dermal fibroblast line from Ku70 and p53 deficient mice (the gift of Dr. P. Hasty, University of Texas Health Sciences Center) was transduced with empty vector (pBABE-puro) retrovirus or a retrovirus expressing mouse Ku70. Substrates were generated by ligating short linkers to the head and tail of a 556 bp linear double-stranded DNA fragment. Linkers possessed 16–17 bp of double-stranded DNA and either 6 or 45 nt 3′ single-stranded overhangs. The linkers with 6 nt overhangs were made by annealing 5′- AGTCTGAGATGGGTGTGAGATCTGC-3′ to 5′-CACTCTCTCACACCCATCTTA-3′ (“head” linker), and 5′-TGACTATACAGCTAAGCGATGATGCAG-3′ to 5′-CATCGCTTAGCTGTATA-3′ (“tail” linker). The linkers with 45 nucleotide 3′ overhangs were generated by annealing 5′-AGTCTGAGATGGGTGTGAGAGTGAAGATCCTCACCTTCGGAGTACTCCTTCTTTTGAGATCTGC-3′ to 5-CTCACACCCATCTCA-3′ (“head” linker) and 5′-TGACTATACAGCTAAGCGATGCTCTCACCGAGCGTATCTGCTGTGTTGTGGATGAATTAGATGCAG-3′ to 5′-CATCGCTTAGCTGTATA-3′ (“tail” linker). Excess linker was removed by QiaQuick purification and substrate purity validated by polyacrylamide gel electrophoresis. 75 ng of substrate was mixed with 1.1 µg of supercoiled pMAX-GFP (Lonza) plasmid carrier and introduced into 2×10^5^ cells in a 10 µl volume by electroporation with one 30 ms 1350 V pulse (Neon, Invitrogen). Cells were harvested after incubation for 1 hour at 37°C, washed, resuspended in Hank's buffered saline solution supplemented with 5 mM MgCl_2_, and extracellular DNA digested by incubation with 6.25 U Benzonase (Novagen) for 15 min at 37°C. Cells were pelleted and DNA purified with the Qiamp kit (Qiagen). Joining efficiency was determined by quantification of head-to-tail junctions by qPCR using primers that either anneal within double-stranded flanks (5′- CTTACGTTTGATTTCCCTGACTATACAG-3′ and 5′- GCAGGGTAGCCAGTCTGAGATG-3′; 6 nt overhang, Figure 5B) or, for the 45 nt overhang substrate only, which anneal to overhang sequence (5′- TAAGCGATGCTCTCACCGAG and 5′- GATGGGTGTGAGAGTGAAGATC; 45 nt overhang, Figure 5C). Results from electroporated samples were further corrected for differences in transfection and sample processing efficiency using a qPCR specific for substrate (5′- GGCACTCTCCAAGGCAAAGA and 5′- ACATGTCTAGCCTATTCCCGGCTT).

### B cell culture and CSR analysis

B cells were isolated from mouse spleens (n = 6 per genotype) and stimulated for class-switching in culture for 72 hr. Where indicated, cultures were incubated with DNA-PKcs inhibitor 20 µM NU7026 (Tocris, Bristol, UK) dissolved in DMSO, or mock-treated. The stimulation procedure and flow-sorting for CSR analysis was as described [31], [67]. Prior to this analysis, cells were counted; numbers and viability were similar for all groups. Sμ-Sγ1 CSR junctions were amplified by PCR using the following conditions for 25 cycles at 95°C (30 s), 55°C (30 s), 68°C (180 s) using the primers (FWD 5′-AATGGATACCTCAGTGGTTTTTAATGGTGGGTTTA-3′; REV 5′ CAATTAGCTCCTGCTCTTCTGTGG-3′) and *Pfu* Turbo (Stratagene, La Jolla, CA). To the PCR reaction, 5 U of *Taq* polymerase (Promega, Madison, WI) was added and incubated at 72°C for 10 min. The resulting product was TOPO TA cloned and transformed into Top10 *E. coli* cells (Life Technologies, Carlsbad, CA) and plasmids were purified and sent for sequencing using M13 FWD and REV primers in addition to the amplification primers for sequencing. 100 clones for each group were analyzed for mutations, deletions, insertions, and sequence overlaps at the junction and both 30 nt upstream and downstream of the junction. p-values were determined by using two-tailed Fisher's exact test.

### Translocation assay

Naïve B cells from three pairs of *Polq^+/+^* and *Polq^−/−^* mice were harvested as above, cultured for 72 hr, and DNA was isolated. 32 separate PCR reactions, each containing the genome from 1×10^5^ cells, was performed with primers to amplify *Myc*/*IgH* translocations and amplified translocations were verified by Southern blotting using internal probes to the *Myc* and *IgH* loci as described [68], [69]. Three independent experiments were performed and the p-value was determined using two-tailed Fisher's exact test. %IgG1 was also measured as an internal control to ensure the B cells from each genotype were switching at a comparable level.

## Supporting Information

Figure S1 Cell sensitivity assays. (A) Clonogenic assay of *Rad51D^+/+^* and *Rad51D^−/−^* CHO cells treated with the indicated doses of olaparib, as a positive control [70]. Colonies were crystal violet stained and counted eleven days later. (B) *Polq^+/+^*, *Polq^−/−^*, *Polq^−/−^*Empty, and multiple clones of *Polq^−/−^*POL and *Polq^−/−^*DM MEF lines were treated with 1 µM bleomycin for 24 hr and cellular ATP levels were measured 72 hr later.
(EPS)

Figure S2 Analysis of DNA double-strand breaks and micronuclei in complemented *Polq* MEFs. Representative immunofluorescence images of *Polq^−/−^*Empty and *Polq^−/−^*WT4 MEF lines stained with DAPI and antibodies against 53BP1 and γH2AX. Scale bar represents 25 µm. Arrows note micronuclei. Pan staining of γH2AX (P), OPT domain staining by 53BP1 (OPT), and examples of colocalization (Co) are noted.
(EPS)

Figure S3 Full-length *chaosI* mutant protein is poorly expressed. 293T cells were transfected with pCDH constructs that contained either the human POLQ cDNA, *ChaosI* mutant (S1977P, corresponding to the S1932P mutated residue in mice), or empty control. (A) 48 hr post transfection lysates were made and immunoblotted with antibodies against FLAG and alpha-Tubulin. (B) Total RNA was isolated from cells and qPCR analysis of POLQ transcript levels were performed using the ΔΔCt method. * denotes non-specific band.
(EPS)

Figure S4 POLQ does not extend a single-stranded oligonucleotide that cannot self-anneal. Increasing amounts of exonuclease-defective *E. coli* polI Klenow fragment denoted as Kf exo- and POLQ were incubated with (A) 5′-^32^P-labeled 20-mer dC oligonucleotide or (B) a 20-mer dC oligonucleotide with a synthetic abasic site on the 3′ end. All reactions include all four deoxynucleotides and were incubated at 37°C for 20 min. The first lane contained no enzyme. The percentage (%) of the product extension from the primer is shown below each lane. * indicates nonspecific band. (C) Model of major 22 nt product formation produced by extension from the primer in Fig. 6B, which has a limited ability to self-anneal.
(EPS)

Figure S5 Absolute quantification of transcript number shows that *Polq* is broadly expressed in tissues. Total RNA was isolated from necropsied C57BL/6 mice by Trizol (Life Technologies). Transcript abundance for *Polq* and DNA polymerase delta catalytic subunit *Pold1* was determined by absolute quantification method as described in the Experimental Procedures. *Gapdh* was used as an internal control to normalize samples.
(EPS)

Figure S6 Mechanism of insertion formation by POLQ during double-strand break repair. After a double-strand break is formed (i), the broken ends are frequently resected enzymatically to form 3′ single-stranded tails (ii). POLQ can extend a 3′ end by templated synthesis from another available DNA strand (iii). This may be the 3′-tailed end near the break, or a DNA strand available at a more distant location, or through possible “snapback synthesis” whereby the tail serves as its own intramolecular template. POLQ has the unique ability to prime DNA synthesis (blue) with minimal base pairing, sometimes slipping in the process (main text Fig. 6). The newly synthesized DNA end then anneals via microhomology to the other 3′ tail at the break (iv), and repair is completed (v) after further DNA synthesis (gray). This process results in an insertion of DNA sequences (blue).
(EPS)

Table S1 POLQ-dependent extension of 3′ DNA ends relies upon homology. Primer extension assays were carried out with a defined substrate (top) incubated in the presence of an active polymerase fragment of POLQ. Products were then incubated with terminal deoxynucleotidyl transferase (TdT) in the presence of only dA, dC, dG, or dTTP to create extension products that terminate with homopolymeric runs. The reaction products were then cloned and sequenced for analysis of extension products. Extension products are listed above for each homopolymeric run.
(XLSX)

Text S1 Additional materials and methods. Expanded methods, including generation of POLQ antibody and POLQ expression constructs, PCR primers, oligonucleotide substrates, and sequencing of DNA extended by POLQ.
(DOCX)
