# Supplementary material for: Mechanism of Suppression of Chromosomal Instability by DNA Polymerase POLQ
Source: PLoS Genet. 2014 Oct 2;10(10):e1004654. doi: 10.1371/journal.pgen.1004654 (PMC4183433; doi:10.1371/journal.pgen.1004654)
Supplement: Text S1 — Additional materials and methods. Expanded methods, including generation of POLQ antibody and POLQ expression constructs, PCR primers, oligonucleotide substrates, and sequencing of DNA extended by POLQ. (DOCX) [file pgen.1004654.s008.docx]

**Additional Materials and Methods**

Generation of POLQ monoclonal antibody

The QL fragment of POLQ (Hogg et al, 2011) was used to immunize a mouse and a monoclonal antibody that recognizes POLQ was generated. The epitope recognized by this antibody (MDACC POLQ20) was mapped using peptide arrays to amino acid residues 2147-2151.

Generation of POLQ expression constructs

The POLQ cDNA was cloned from pFastbacHTc/POLQ ([Seki et al., 2003](#_ENREF_2)) using KOD Xtreme (EMD Millipore, Billerica, MA) polymerase using the following primers and conditions (FWD 5’ CGGGATCCTAGCACCATGAATCTTCTGCGTCGGAGTG  3’, REV 5’-CACATCAAAGTCCTTTAGCTCTCCCGCCGGCGTTTTCCTTTT-3’); 20 cycles at 95 °C (30 s), 60 °C (30 s), 68 °C (10 min). PCR products were subsequently digested with *BamH*I and *Not*I and ligated into similarly digested pBluescript SKII+ vector. pBluescript/POLQ was propagated and sequenced to ensure no mutations, before the POLQ cDNA was digested with the aforementioned restriction enzymes and ligated into pCDH-FHC vector. The final vector pCDH-FHC/POLQ was propagated and sequenced for insert verification and mutation analysis. Engineering of the mutant pCDH/POLQ vectors is detailed in the supplemental methods section.

The individual domains of POLQ (Helicase-like, Central, and Pol domain) were amplified from pBluescript/POLQ using KOD Xtreme and the following primers before being digested with *Bam*HI and *Not*I, then ligated into pBluescript:

Helicase-like (HLD) domain

CDH HLD5’ CGGGATCCTAGCACCATGAATCTTCTGCGTCGGAGTG;

CDH HLD3’ TGCCCATGCGGCCGCGATTATAAAACTCTCGAGCAATGATATCTGC

Central (CEN) domain

CDH CEN5’ AGGAAAGGATCCATTGCTCGAGAGTTTTATAATCTACATCATCAAGC; CDH CEN3’ TCGTTGCGGCCGCTTCCTTTCGCAAGCAAGATTGAAGGTACCACATC

Polymerase (POL) domain

CDH POL5’ AGGAAAGGATCCGATGTGGTACCTTCAATCTTGCTTGCGAAAGGAA;

CDH POL3’ AAAAGGAAAagcggccgcCACATCAAAGTCCTTTAGCTCTCC

Vectors were sequenced to verify no mutations before site-directed mutagenesis was performed with the following plasmid-primer combinations using KOD Xtreme:

*POLQ*-K121M

pBluescript/HLD

Forward: 5' CTACAAGTGCTGGGATGACTCTTGTGGCAG 3' ;

Reverse: 5' CTGCCACAAGAGTCATCCCAGCACTTGTAG 3'

*POLQ-D2330A/Y2331A*

pBluescript/Pol

Forward: 5' GTTCAATACTGGCTGCTGCCGCCTCTCAGCTTGAACTGAG 3' ;

Reverse: 5' CTCAGTTCAAGCTGAGAGGCGGCAGCAGCCAGTATTGAAC 3'

*POLQ-S1977P*

PBluescript/Pol

Forward: 5' CTATAAAATTCTTCTTCTTCCTTGTGGCATCTCCTTGGAG 3';

Reverse: 5' CTCCAAGGAGATGCCACAAGGAAGAAGAAGAATTTTATAG 3'

Following mutational verification, the individual domains were digested with the corresponding restriction enzymes before being ligated into pCDH-FHC that was previously digested with *BamH*I and *Not*I:

pBluescript/HLD (*BamH*I, *Xho*I)

pBluescript/CEN (*Xho*I, *Kpn*I)

pBluescript/POL (*Kpn*I, *Not*I)

Following ligation, plasmids were sequenced to ensure proper ligation and maintenance of engineered mutations.

Oligonucleotide Substrates

Gel purified primer oligonucleotides 16-1 and PA42 were purchased from Bio-Synthesis, C20 was from Midland, C19+THF was from IDT. 16-1, C20, C19+THF were 5'-labeled using polynucleotide kinase (Promega) and [γ-^32^P] dATP (PerkinElmer). 5'-[^32^P]-labeled 16-1 primer was annealed to PA42 to generate a double stranded DNA substrate as described ([Takata et al., 2006](#_ENREF_3))

16-1 (primer)+PA42 (template) substrate:

5'-^32^P-CACTGACTGTATGATG-3'

3'-GTGACTGACATACTACTTCTACGACTGCTC-5'

16-1 substrate (primer only):

5'-^32^P-CACTGACTGTATGATG-3'

C20 substrate (primer only):

5'-^32^P-CCCCCCCCCCCCCCCCCCCC-3'

C19+THF (tetrahydrofuran, an AP site analog) substrate (primer only):

5'-^32^P-CCCCCCCCCCCCCCCCCCC[THF]-3'

Sequencing of DNA extended by POLQ

A truncated version of human POLQ (residues 1792-2590) was expressed in *E. coli* and purified as described ([Hogg et al., 2011](#_ENREF_1)). Oligonucleotides were purchased from Eurofins MWG operon (Germany).

The 200 μl primer extension reaction contained: [20mM Tris-HCl (pH 8.8), 4% glycerol, 10 mM MgCl_2_, 0.1 mM EDTA, 80 μg/ml bovine serum albumin (BSA), 10 pmol of the tetrachlorofluorescein (TET) labelled oligonucleotide “no-hairpin” (5’-(TET)-GTTTGTGAGTTTCC-3’) and 1.25 μg POLQ_(1792-2590)_]. The primer extension reaction was incubated at 22^o^C for two hours and terminated by the addition of 10 μl 0.5 M EDTA and POLQ was heat inactivated for one min at 100^o^C. As a control 10 μl of the reaction mixture was separated on a polyacrylamide sequencing gel and the amount of POLQ extended products was evaluated with a Typhoon 9400 Variable Mode Imager (GE Healthcare). The remaining DNA was precipitated overnight at -20^o^C with 4 μg carrier tRNA, 4 μg glycogen, 0.25 volumes of 5 M ammonium acetate and 2.5 volumes of ethanol. Precipitated DNA was dissolved in 100 μl 10mM Tris-HCl (pH 8.0) and used as template for terminal nucleotidyl transferase (TdT) (Thermo Scientific). 40 μl TdT reactions contained 20 μl POLQ extended oligonucleotide, 1X TdT buffer, 3.2 μM either of dATP/dTTP or 1.5 μM either of dCTP/dGTP and 30 U TdT. The reactions were incubated at 37^o^C for 15 min followed by heat inactivation of TdT at 70^o^C for 10 min. In control experiments TdT reactions were performed with no-hairpin oligonucleotide, which had not been extended by POLQ, as template. 10 μl from each reaction was separated on a polyacrylamide sequencing gel and the amount of TdT extended products was visualized and evaluated with a Typhoon 9400 Variable Mode Imager. POLQ + TdT extended no-hairpin DNA and only-TdT extended no-hairpin DNA were amplified by polymerase chain reaction (PCR). 25 μl PCR reactions contained: 0.2 μl from respective TdT reaction, 5 pmol of the oligonucleotide EcoRI-nohairpin (5’- TTTTGAATTCGTTTGTGAGTTTCC-3’), 5 pmol of either HindIII-25A (5’-TTTTAAGCTTAAAAAAAAAAAAAAAAAAAAAAAAA-3’), HindIII-25T-R (5’-TTTTAAGCTTTTTTTTTTTTTTTTTTTTTTTTTTT-3’), HindIII-15C-R (5’-TTTTAAGCTTCCCCCCCCCCCCCCC-3’) or HindIII-15G-R (5’-TTTTAAGCTTGG­GGGGGGGGGGGGG-3’), 1X GoTaq buffer, 200 µM of each dNTP and 1 U GoTaq DNA polymerase (Promega). PCR cycling conditions were: five cycles (94^o^C 30 s, 50^o^C 30 s and 72^o^C 10 s) followed by 30 cycles (94^o^C 30 s, 55^o^C 30 s and 72^o^C 10 s). PCR reactions were purified with QIAquick Nucleotide Removal Kit (Qiagen, Germany) and digested with *EcoR*I and *Hind*III. Digested PCR products were then separated on a 2% agarose gel containing Gel Green Nucleic Acid Stain. DNA was excised, purified with QIAquick Gel Extraction Kit (Qiagen, Germany), ligated into an *EcoR*I-*Hind*III digested pUC18 vector and transformed into competent XL1-Blue bacterial cells. Plasmid containing bacterial clones were selected for on LB plates containing 100 µg/ml carbenicillin and plasmids were isolated with the E-Z 96 fastfilter plasmid kit (Omega bio-tek) and sent for sequencing (Eurofins MWG Operon, Germany).

**Supplemental References**

Hogg, M., Seki, M., Wood, R.D., Doublié, S., and Wallace, S.S. (2011). Lesion bypass activity of DNA polymerase theta (POLQ) is an intrinsic property of the pol domain and depends on unique sequence inserts. J Mol Biol 405, 642-652.

Seki, M., Marini, F., and Wood, R.D. (2003). POLQ (Pol θ), a DNA polymerase and DNA-dependent ATPase in human cells. Nucleic Acids Res 31, 6117-6126.

Takata, K.I., Shimizu, T., Iwai, S., and Wood, R.D. (2006). Human DNA polymerase N (POLN) is a low-fidelity enzyme capable of error-free bypass of 5S-thymine glycol. J Biol Chem 281, 23445-23455.
